# High-volume lactated Ringer’s solution with human albumin *versus* standard-volume infusion as a prophylactic treatment for post-endoscopic retrograde cholangiopancreatography pancreatitis: randomized clinical trial

**DOI:** 10.1093/bjsopen/zrae149

**Published:** 2025-01-21

**Authors:** Ekaphan Shatsnimitkul, Issaree Laopeamthong, Amarit Tansawet, Suphakarn Techapongsatorn, Wisit Kasetsermwiriya, Poramet Leungon, Pakkapol Sukhvibul

**Affiliations:** Department of Surgery, Faculty of Medicine Vajira Hospital, Navamindradhiraj University, Bangkok, Thailand; Department of Surgery, Faculty of Medicine Vajira Hospital, Navamindradhiraj University, Bangkok, Thailand; Department of Research and Medical Innovation, Faculty of Medicine Vajira Hospital, Navamindradhiraj University, Bangkok, Thailand; Department of Surgery, Faculty of Medicine Vajira Hospital, Navamindradhiraj University, Bangkok, Thailand; Department of Surgery, Faculty of Medicine Vajira Hospital, Navamindradhiraj University, Bangkok, Thailand; Department of Surgery, Faculty of Medicine Vajira Hospital, Navamindradhiraj University, Bangkok, Thailand; Department of Surgery, Faculty of Medicine Vajira Hospital, Navamindradhiraj University, Bangkok, Thailand

## Abstract

**Background:**

Adverse events after endoscopic retrograde cholangiopancreatography (ERCP) are rare, and post-ERCP pancreatitis is a serious adverse event. This study aimed to determine the role of aggressive intravenous hydration with lactated Ringer’s solution at a specific volume with 20% human albumin before ERCP in reducing the incidence of post-ERCP pancreatitis.

**Methods:**

This study was a single-centre randomized clinical trial. The participants were randomly assigned to two groups: those who received aggressive intravenous hydration with 20% human albumin and lactated Ringer's solution (intervention group), and those who received standard-volume intravenous hydration with lactated Ringer's solution (control group). The primary endpoint was post-ERCP pancreatitis. Participants and outcome assessors were blinded to treatment allocation. Comparison was performed using the chi-square, the Fisher’s exact, the Student’s *t*, or the Mann–Whitney U tests, where appropriate.

**Results:**

Of 300 randomized participants, 149 and 144 participants from the intervention and control group were included in the analysis. There was no significant difference in the post-ERCP pancreatitis rate (*n* = 10; 6.7% *versus n* = 9; 6.3%, *P* = 0.873) between the intervention and control groups. High-risk procedures (that is pancreatic duct wiring, pancreatic duct injection, precut sphincterotomy, and balloon dilation of the ampulla) were significantly associated with post-ERCP pancreatitis compared with low-risk procedures (*n* = 15; 15% *versus n* = 4; 2.1%, *P* < 0.001). In the high-risk procedures population, the intervention and control groups had increased post-ERCP pancreatitis rates (*P* = 0.716). Two participants in each group developed pulmonary congestion.

**Conclusion:**

Aggressive peri-ERCP intravenous hydration with lactated Ringer's solution combined with 50 ml of 20% human albumin did not prevent post-ERCP pancreatitis. None of the subgroups presented with prophylactic effects.

**Trial registration:**

Thai Clinical Trials Registry (TCTR20240405003)

## Teaser

This study aimed to determine the role of aggressive intravenous hydration with lactated Ringer’s solution (LRS) at a specific volume with 20% human albumin before ERCP in reducing the incidence of post-ERCP pancreatitis (PEP). The participants were randomly assigned to two groups: those who received aggressive intravenous hydration with 20% human albumin and LRS (intervention group), and those who received standard-volume intravenous hydration with LRS (control group). There was no significant difference in the PEP rate between the intervention and control groups.

## Introduction

Pancreatitis is the most frequent complication after endoscopic retrograde cholangiopancreatography (ERCP), with an incidence rate of 3.5–9.7%^1,2^, and a mortality rate ranging from 0.1% to 0.7%^1,2^.

Several interventions can reduce the incidence of post-ERCP pancreatitis (PEP). These include rectal non-steroidal anti-inflammatory drugs (NSAIDs)^3,4^, pancreatic duct stenting^5,6^, and aggressive peri-procedural hydration^7,8^, but NSAIDs are not always available in suppository form and pancreatic duct stenting can be performed only when pancreatic duct cannulation can be achieved.

Early aggressive hydration is recommended for acute pancreatitis in many guidelines^9,10^. The study of Wu *et al.*^11^ on patients with acute pancreatitis showed that lactated Ringer’s solution (LRS) was associated with a low incidence of systemic inflammatory response syndrome and a low level of C-reactive protein compared with normal-volume saline resuscitation. In a recent randomized clinical trial (RCT)^12^, no benefit of aggressive fluid resuscitation was found in acute pancreatitis patients, whereas rates of fluid overload were much higher than the moderate fluid resuscitation group. For PEP prophylaxis, LRS became the most investigated intravenous fluid, as confirmed in several RCTs^7,8,13–15^.

Because aggressive volume infusion may lead to fluid overload and pulmonary congestion, it might be safer to reduce the volume of crystalloid solution. The current study aimed to investigate the efficacy of adding a colloid solution to LRS, preserving the prophylactic effect of aggressive intravenous hydration without increasing the risk of fluid overload.

## Methods

### Study design and setting

This study was a parallel-arm RCT conducted at a single university hospital where approximately 200 cases of ERCP are performed annually. The patients were enrolled from January 2022 to July 2023. This study was approved by the institutional ethics committee and was conducted in accordance with the Belmont Report and Declaration of Helsinki. It was also approved by the Vajira ethics committee before enrolment (COA237/2564). The participants and outcome assessors were blinded to treatment allocation. Patients included were aged 18 years or older, with a pancreaticobiliary disease requiring endoscopic intervention, and with a written informed consent. The exclusion criteria were patients with a history of congestive heart failure according to the New York Heart Association class 2 and those with signs of volume overload including pulmonary congestion and peripheral oedema, or with significant co-morbidities (that is chronic kidney disease (CKD) stage 3 or higher, chronic obstructive pulmonary disease, and decompensated cirrhosis), active pancreatitis, unstable haemodynamics, and allergy to albumin. Please see Supplementary Materials for full study protocol.

### Interventions

**Fig. 1:**
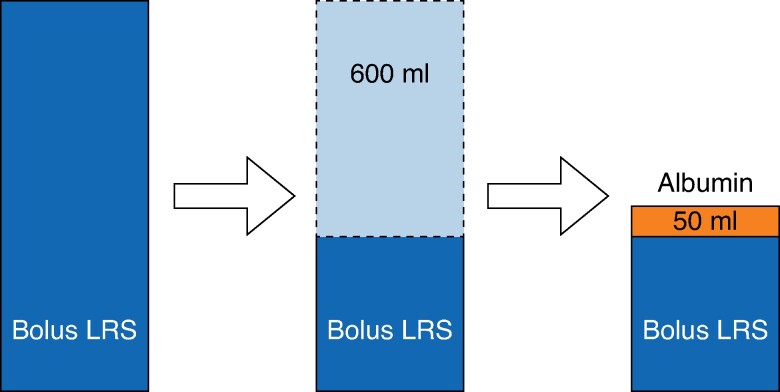
Bolus intravenous fluid in the intervention group (LRS, lactated Ringer's solution)

Before ERCP, bolus IV fluid was administered to the intervention group, equivalent to 15 ml/kg of LRS. Whereas LRS 1000 ml can raise a plasma volume of 300 ml^16^, 20% human albumin 50 ml results in 200 ml plasma volume expansion^17^. Comparing plasma volume expansion from LRS to 20% human albumin, approximately 600 ml of the calculated LRS volume is almost equal to and was replaced with 50 ml of 20% human albumin. The 20% human albumin was administered on the ward approximately 1 h before the procedure, followed by the rest of the bolus LRS (*Fig. 1*). After the bolus dose, LRS was administered at a rate of 3 ml/kg/h until 8 h post-ERCP, followed by the maintenance rate. In the control group, LRS was administered at a fixed rate of 1.5 ml/kg/h, starting approximately at 1 h before ERCP. If the participant’s body weight exceeded 80 kg, the ideal body weight was used for fluid volume calculation.

In case of PEP occurrence, a high-volume LRS was administered regardless of the allocation group. ERCP was performed under general anaesthesia and the procedure started with selective bile duct cannulation, followed by complete cholangiography. Sphincterotomy was routinely conducted in a naïve ampulla. In case of inadvertent pancreatic duct cannulation, a pancreatic duct stent was routinely inserted. Contrast media used for cholangiography were withdrawn as much as possible before termination. Prophylactic antibiotics were administered before ERCP in all participants if antibiotics were not administered based on the therapeutic indication (third-generation cephalosporins if no contraindication). None of the participants received prophylactic rectal NSAIDs.

### Outcomes

The primary endpoint was PEP that occurred within 24 h after ERCP. A PEP diagnosis was made based on new-onset abdominal pain consistent with acute pancreatitis plus elevated pancreatic enzyme (that is amylase or lipase) levels more than three times the upper normal limit. The secondary outcome was high pancreatic enzyme level, which was measured at approximately 4 and 24 h after the procedure. Significant elevation was defined as a serum level that was three times higher than that of the upper normal limit (that is >300 U/l for amylase and >180 U/l for lipase). Signs of fluid overload (dyspnoea with crackles in chest auscultation confirmed with chest radiography, and peripheral oedema) and ERCP-related complications were recorded. Clinical symptoms and signs were ascertained every 6 h by a physician blinded to the treatment allocation.

### Randomization

Random sequences of treatment allocation were generated in STATA version 17 (StataCorp, Texas, USA) using the permuted block randomization technique. The block size varied between four and eight participants with a ratio of 1:1 between the intervention and control groups. Sequentially numbered, opaque, sealed envelopes were used for concealment. Random sequences and sealed envelopes were prepared by a statistician who was not involved in this trial.

### Sample size determination

A previous RCT^14^ compared high- *versus* standard-volume LRS administered during the peri-ERCP period and showed that the incidence rates of PEP were 3% and 11.6% in the high- and standard-volume groups respectively. The current study was assumed to have the same PEP risk as the previous study (that is 3% and 11.6%). A two-sided significance level was applied, for which type I error, power, and randomization ratio were set at 5%, 80%, and 1:1 respectively. As a result, 286 participants were required for the current study.

### Statistical analysis

All analyses were performed in a modified intention-to-treat (ITT) manner, where participants belonged to the groups to which they were randomized but were excluded if they did not undergo ERCP (that is no PEP risk). Data were described as frequency and percentage if categorical variables and as mean and standard deviation or median and interquartile range if continuous variables. The baseline and procedure-related characteristics and the outcome variables were compared using the chi-square test or the Fisher’s exact test for categorical variables and the independent Student’s *t*-test or the Mann–Whitney U test for continuous variables.

Risk ratios (RRs) along with 95% confidence intervals were further computed using Altman’s formula^18^. Regarding clinical context^19,20^, a subgroup of patients undergoing a high-risk procedure (that is pancreatic duct wiring, pancreatic duct injection, precut sphincterotomy, and balloon dilation of the ampulla) or having naïve ampulla were analysed. *P* ≤ 0.05 from a two-sided statistical test was considered significant. All analyses and sample size estimation were performed using STATA version 17 (StataCorp, Texas, USA).

## Results

### Baseline characteristics

**Fig. 2:**
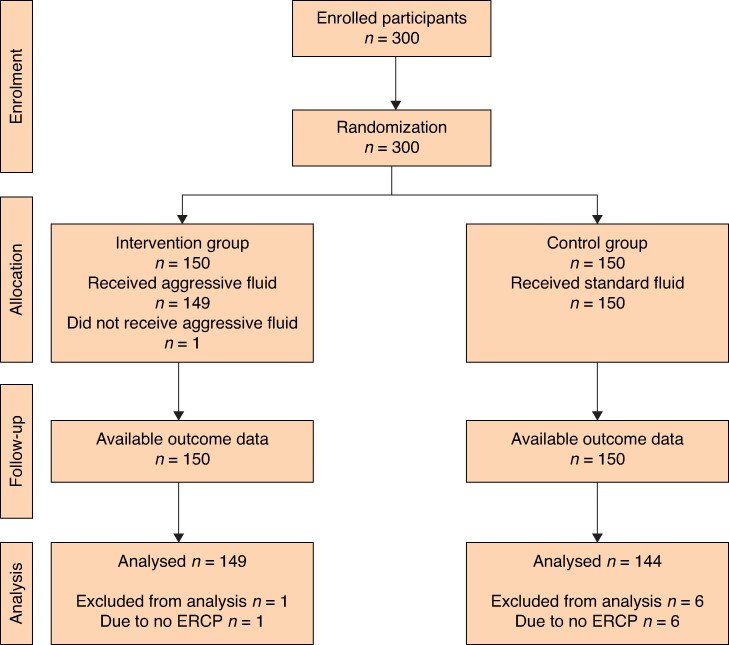
Study flow diagram. ERCP, endoscopic retrograde cholangiopancreatography.

Three hundred participants were randomized. After excluding participants who did not undergo ampulla cannulation, 293 participants were included in the modified ITT analysis (*Fig. 2*). Only one participant from the intervention group who did not receive intravenous albumin violated the study protocol, but was still included in the intervention group to comply with the modified ITT analysis. The mean(s.d.) age of the participants was 54.6(15.5) years and 55% (*n* = 161) were women. The underlying conditions in 17.4% (*n* = 51) and 39.6% (*n* = 116) of the patients were diabetes and hypertension respectively. Only a few participants (*n* = 5) had stage I–II CKD. A total of 8.7% (*n* = 13) and 9.7% (*n* = 14) of the participants in the intervention and control groups respectively had a history of pancreatitis (*Table 1*).

**Table 1:** Participant characteristics

Baseline characteristics	Intervention group (*n* = 149)	Control group (*n* = 144)	*P*
Age (years), mean(s.d.)	54.5(15.8)	54.6(15.3)	0.952
**Sex**			
Female	82 (55)	79 (54.9)	0.976
Weight (kg), mean(s.d.)	62.5(11.1)	61.6(11.8)	0.459
BMI (kg/m^2^), median (i.q.r.)	23.5 (20.7–25.7)	23 (20.3–26.4)	0.970
Diabetes	28 (18.8)	23 (16)	0.525
Hypertension	64 (43)	52 (36.1)	0.231
CKD stage I–II	4 (2.7)	1 (0.7)	0.371
History of pancreatitis	13 (8.7)	14 (9.7)	0.768
Antiplatelets/anticoagulants	9 (6)	5 (3.5)	0.413
**Pathology**			
Cholelithiasis	99 (66.4)	99 (68.8)	0.618
Peri-ampullary neoplasm	7 (4.7)	9 (6.3)	
Hilar and mid CBD neoplasm	22 (14.8)	13 (9)	
Benign biliary stricture	6 (4)	9 (6.3)	
Bile leakage	9 (6)	10 (6.9)	
Chronic pancreatitis	6 (4)	4 (2.8)	
Previous ERCP	54 (36.2)	61 (42.4)	0.284
**Procedure-related risk**			
Pancreatic duct wiring	40 (26.9)	29 (20.1)	0.971
Pancreatic duct injection	3 (2)	1 (0.7)	
Precut sphincterotomy	8 (5.4)	14 (9.7)	
Balloon ampulla dilation	13 (8.7)	14 (9.7)	
Biliary stenting	56 (37.6)	52 (36.1)	0.794
Pancreatic duct stenting	30 (20.1)	25 (17.4)	0.543
Through-the-scope cholangioscopy	14 (9.4)	18 (12.5)	0.394
Electrohydraulic lithotripsy	8 (5.4)	18 (12.5)	0.032
Total procedure time (min), median (i.q.r.)	30 (30–52)	30 (30–51.5)	0.858

Values are *n* (%) unless otherwise indicated. BMI, body mass index; CBD, common bile duct; CKD, chronic kidney disease; ERCP, endoscopic retrograde cholangiopancreatography.

Cholelithiasis (*n* = 198) was the leading pathology, followed by hilar and common bile duct neoplasm (*n* = 35), bile leakage (*n* = 19), periampullary neoplasm (*n* = 16), benign stricture (*n* = 15), and chronic pancreatitis (*n* = 10). A total of 36.2% (*n* = 54) and 42.4% (*n* = 61) of the intervention and control groups respectively had a previous history of ERCP. The median procedure time was 30 (i.q.r. 30–52) minutes. The participants did not significantly differ in terms of characteristics (*Table 1*). No statistical difference was observed between the study groups in terms of procedure-related risks and additional interventions, except electrohydraulic lithotripsy (*P* = 0.032). The median volumes of pre-ERCP LRS were 600 (i.q.r. 460–770) and 90 (i.q.r. 80–100) ml in the intervention and control groups respectively (*P* < 0.001). The mean(s.d.) total 24-h LRS volumes were 3375.6(952.5) ml in the intervention group and 2342.4(722.3) ml in the control group (*P* < 0.001). Only 19 (6.5%) participants denied having laboratory examination at 24 h after procedure and recovered uneventfully.

### Clinical outcomes

The PEP rates did not significantly differ between the intervention and control groups (*n* = 10 *versus n* = 9, *P* = 0.873, RR 1.07 (95% c.i. 0.45 to 2.57); *Table 2*) and there were no severe pancreatitis cases. The two groups did not significantly differ in terms of pancreatic enzyme elevation, with *P* = 0.773 (RR 0.93 (95% c.i. 0.56 to 1.53)) and 0.478 (RR 0.86 (95% c.i. 0.58 to 1.29)) for serum amylase and lipase respectively. Two participants in each group developed pulmonary congestion that required prolonged hospital stay and two participants had bleeding requiring re-endoscopy, one of whom in the control group developed pulmonary congestion from aggressive fluid resuscitation.

**Table 2:** Comparison of pancreatitis and pancreatic enzyme elevation between study groups

Outcome	Intervention group (*n* = 149)	Control group (*n* = 144)	*P*
PEP	10 (6.7)	9 (6.3)	0.873
Serum amylase elevation*	25 (16.8)	26 (18.1)	0.773
Serum lipase elevation*	34 (22.8)	38 (26.4)	0.478

Values are *n* (%). *Elevation > 3 times of upper limit of normal value at 4 or 24 h after procedure. PEP, post-ERCP pancreatitis; ERCP, endoscopic retrograde cholangiopancreatography.

The PEP rates of intervention and control groups of 100 patients who had undergone a high-risk procedure were (*n* = 7) 13.7% and (*n* = 8) 16.3% respectively, but the difference was not statistically significant (*P* = 0.716, RR 0.84 (95% c.i. 0.33 to 2.14)). The incidence of PEP did not significantly differ between the intervention and control groups among 178 naïve ampulla participants (*n* = 8 *versus n* = 6, *P* = 0.768, RR 1.16 (95% c.i. 0.42 to 3.22)).

No significant association was observed between PEP and participant-related factors, including age <40 years (*P* = 0.626, RR 1.28 (95% c.i. 0.48 to 3.41)), female sex (*P* = 0.790, RR 1.13 (95% c.i. 0.47 to 2.72)), and history of pancreatitis (*P* = 0.838, RR 1.16 (95% c.i. 0.28 to 4.75)). Electrohydraulic lithotripsy, which was imbalance between treatment groups, was not associated with PEP (*P* = 0.075, RR 2.74 (95% c.i. 0.98 to 7.65)). Only high-risk procedures were significantly associated with PEP (*n* = 15 *versus n* = 4, *P* < 0.001, RR 7.24 (95% c.i. 2.47 to 21.23)).

## Discussion

This study showed that aggressive intravenous hydration with LRS combined with human albumin was not beneficial when used as a PEP prophylaxis. The incidence of pulmonary congestion between the intervention and control groups did not differ.

The study of Park *et al*.^14^ demonstrated a significant reduction in PEP incidence in the aggressive fluid hydration group (3%) compared with the standard-volume hydration group (11.6%). Based on these results, only 286 participants were required for the current study. Given results from other large-scale RCTs^13,15^, the number of participants could be 678. If the enrolment was planned for 678 participants and the results from the current study were considered as the interim analysis, a *P* of 0.873 from 293 participants had already passed the O’Brien and Fleming futility boundary^21^. In other words, significance was unlikely to be indicated by enrolling more participants.

The current study included both naïve ampulla and previous ERCP patients, whereas other trials were restricted to patients who had never undergone ERCP before. This difference might explain the null results, which were not observed in other studies^13–15^. Even though subgroup analysis was conducted in the naïve ampulla participants, aggressive fluid hydration with albumin was not beneficial compared with standard-volume infusion in the current study. The aggressive fluid hydration with albumin also had no effect in the high-risk group, unlike the results of the Park *et al*. study^14^. The PEP incidence was two times higher than average although there was utilization of pancreatic duct stents.

The substantial difference between the current study and the others^13–15^ is the bolus fluid loading, replaced with 20% human albumin in the current trial. The results revealed that an infusion with 50 ml of 20% human albumin did not prevent PEP, suggesting that only the volume of the bolus LRS was important. As far as is known, no study has investigated the effect of colloid solution in PEP prophylaxis and the benefits of them or 20% albumin at higher volumes are still inconclusive.

The control intervention in the current study was a standard-volume infusion, so the difference in fluid overload incidence with the recommended hyperhydration regimen could not be evaluated. Unlike acute pancreatitis, recent meta-analysis^22^ has indicated that rates of fluid overload were not significantly increased by aggressive hydration in prophylactic indication.

The strength of the current study was its randomization design where most baseline characteristics were well balanced. This study also had limitations. The variation in the combination of 20% human albumin and LRS could not be evaluated because only two parallel groups were created. Endoscopists were not blinded to the treatment allocation, and to decrease possible bias, the study protocol in terms of ERCP technique, co-medication, and outcome assessment was set in advance. The current study enrolled a lower number of participants compared with other trials^13,15^. Small but significant effects would not be detected, but this possibility should be extremely low considering the futility boundary discussed earlier.

The current study did not recommend partial replacement of LRS with 20% human albumin for PEP prophylaxis, especially in general ERCP patients. As indicated in the previously mentioned guideline^22^, LRS alone would be effective to prevent PEP without increasing harm. Due to this fact, conducting further studies to examine the effect of colloid solutions would be irrational.

In conclusion, aggressive peri-ERCP fluid hydration with LRS combined with 50 ml 20% human albumin did not prevent PEP in general patients undergoing ERCP. Based on the current study, the combined 20% human albumin and LRS might not have a prophylactic effect in any of the subgroups.

## Supplementary Material

zrae149_Supplementary_Data

## Data Availability

Access to the data set from this study is available from the corresponding author upon reasonable request and approval.
