# Supplementary material for: High-volume lactated Ringer’s solution with human albumin versus standard-volume infusion as a prophylactic treatment for post-endoscopic retrograde cholangiopancreatography pancreatitis: randomized clinical trial
Source: BJS Open. 2025 Jan 21;9(1):zrae149. doi: 10.1093/bjsopen/zrae149 (PMC11749547; doi:10.1093/bjsopen/zrae149)
Supplement: zrae149_Supplementary_Data [file zrae149_supplementary_data.zip › Supplementary-Material.docx]

**High-volume lactated Ringer’s solution with human albumin versus standard-volume infusion as a prophylactic treatment for post-endoscopic retrograde cholangiopancreatography pancreatitis: randomised controlled trial**

Ekaphan Shatsnimitkul^1,*^; Issaree Laopeamthong^1,*^; Amarit Tansawet^2^; Suphakarn Techapongsatorn^1^; Wisit Kasetsermwiriya^1^; Poramet Leungon^1^; Pakkapol Sukhvibul^1^

^1^ Department of Surgery, Faculty of Medicine Vajira Hospital, Navamindradhiraj University, Bangkok, Thailand

^2^ Department of Research and Medical Innovation, Faculty of Medicine Vajira Hospital, Navamindradhiraj University, Bangkok, Thailand

^*^ Equal contribution

**Corresponding author:**

Pakkapol Sukhvibul, MD

Department of Surgery, Faculty of Medicine Vajira Hospital, Navamindradhiraj University, Bangkok, Thailand

681 Samsen Road, Dusit, Bangkok, Thailand

Email: [pakkapol@nmu.ac.th](mailto:pakkapol@nmu.ac.th), Tel: +66-2244-3282

**Supplementary Materials - Index**

**Protocol** page 2

**References** page 7

**Protocol**

**Study objective**

***Primary objective:*** To compare rates of post-ERCP pancreatitis (PEP) between the intravenous albumin-administered plus high-volume lactated Ringer solution (LRS) group and standard LRS infusion group

***Secondary objectives:***

1. To compare rates of pancreatic enzyme elevation between the intravenous albumin-administered plus high-volume LRS group and standard LRS infusion group
2. To compare rates of fluid overload between the intravenous albumin-administered plus high-volume LRS group and standard LRS infusion group

**Study design and setting**

***Design:*** Parallel-arm randomised controlled trial (RCT) – participants and outcome assessors blined

***Setting:*** Vajira Hospital (Navamindradhiraj University)

**Inclusion criteria**

- Age ≥ 18 years
- Have pancreatobiliary diseases requiring ERCP
- Willing to participate

**Exclusion criteria**

- Have a history of congestive heart failure
- Have signs of fluid overload
- Suffer significant comorbidities
  - CKD stage ≥ 3
  - Decompensated cirrhosis
  - COPD
- Suffer active pancreatitis
- Unstable hemodynamics
- Allergy to human albumin

**Intervention**

Bolus fluid will be calculated to have a volume of 15 ml/kg of LRS. Then, 600 ml of this volume will be replaced with 50 ml of 20% human albumin^1,2^ and started ~ 1 h prior to procedure. The rest of the bolus fluid will be given subsequently. LRS will be administered at a rate of 3 ml/kg/h until 8 h post-ERCP, followed by the maintenance rate. Ideal body weight will be used in fluid calculation (for both intervention and control groups) if pariticipants weigh > 80 kg.

**Control**

LRS will be administered at a fixed rate of 1.5 mL/kg/h and started approximately at 1 h before ERCP.

**Co-interventions**

***ERCP procedure:***

ERCP will be performed under general anesthesia with prone or supine position depended on endoscopist preference. Steps of the procedure include selective bile duct cannulation, sphincterotomy, complete cholangiography, and therapeutic interventions regarding pathology. These steps can be modified and varied between cases as needed. Before termination, the rest of contrast media will be removed as much as possible. All participants will be discharged home after 24 h if uneventful.

***Antibiotic:***

Prophylactic antibiotic will be the 3^rd^ generation cephalosporins – ceftriaxone – if there is no history of drug allergy. Participants who require antibiotic for therapeutic indications will receive empirical antibiotics such as cephalosporins, carbapenams, or piperacillin/tazobactam and de-escalate later as indicated.

***Other treatments:***

Treatment will be reconciled with participant’s underlying conditions. If adverse events developed, treatments would be delivered regarding a standard guideline. This includes aggressive intravenous fluid in case of PEP.

**Outcomes and measurement**

***Primary outcome:***

PEP will be diasnosed if participants develop acute epigastrium pain within 24 h after ERCP accompanying with elevated serum amylase or lipase levels. Definition of elevated pancreatic enzyme is a serum level > 3 times higher than the upper normal limit (>300 U/L for and >180 U/L for amylase and lipase, respectively). Signs and symptoms will be evaluated every 6 h until 24 h after ERCP by physicians blinded to allocation. Blood will be drawn for pancreatic enzyme measurement at 4 and 24 h after ERCP. This outcome will be analysed as a binary endpoint.

***Secondary outcome:***

1. Elevated serum pancreatic enzyme level: will be measured at 4 and 24 h after ERCP. Definition of this outcome is described as above. This outcome will be analysed as a binary endpoint (the occurrence of elevated enzyme level within 24 h after ERCP).
2. Fluid overload: any dyspnea with crackle sounds from chest auscultation and peripheral edema will be recorded. This outcome will be analysed as a binary endpoint.
3. Other complications and mortality: the number of each event will be records separately.

**Consent**

Patients required ERCP will be admitted to the hospital. Researchers will screen for eligible patients and invite to participate in this study. Details of the study including rationales, benefits, and risks will be thoroughly informed. Patients will be informed that their decision will not affect on their treatments, and they can withdraw from the study at any time. Written informed consent will be obtained from participants who are willing to participate.

**Randomisation and concealment**

A permuted block randomisation with varying block size (4-8 participants per block), 1:1 allocation ratio, will be used to generate random sequence list. This sequence will be concealed with sequentially numbered, opaque, sealed envelopes, which will be revealed during admission at 1 day before ERCP. Random sequence list and envelopes will be prepared by statisticians who will not involve in this study.

**Blinding**

Participants and outcome assessors will be blinded to the treatment allocation. Also, physician team, except endoscopists, will be blinded. Physician team will be unblinded in case a knowledge of treatment allocation is necessary, for example, PEP.

**Data management**

Participants will be anonymised and identified via assigned study ID. No participant’s identification will appear in data record form (DRF). Research assistants will record participant’s data in DRF. A principal researcher will check for DRF completeness before database entry. Paper documents will be kept in a safe locked cabinet and will be destroyed after 5 years.

**Sample size estimation**

The Park et al’s study^3^ has indicated that rates of PEP in aggressive and standard volume intravenous fluid was 3% and 11.6%, respectively. Based on these findings, 286 participants would be enrolled for the current study given alpha-error, power, and allocation ratio of 5%, 20%, and 1:1, respectively. To compensate for enrolment error that might occur, 300 participants will be recruited.

**Statistical analysis**

Descriptive statistics will be conducted to describe data characteristics. Continuous data will be described with mean and standard deviation (SD) or median and interquartile range (IQR) depended on whether their distribution is normal or not. Categorical data will be described with percentage.

Comparison between groups will be performed by statistical test as follows: the chi-square or Fischer’s exact tests for categorical data (e.g. the outcome data), and the independent student t- or Mann-Whitney U tests for continuous data (normal and non-normal distribution, respectively). Risk ratios and their 95% confidence intervals, representing the strength of association between the factors of interest and the outcomes, will be estimated. A P-value of ≤ 0.05 will be considened as statistical significance.

Subgroup analysis will be conducted for naïve ampulla participants and participants who undergo high-risk procedures defined by pancreatic duct wiring, pancreatic duct injection, precut sphincterotomy, and balloon dilation of the ampulla.

**Monitoring and safety**

Any adverse events and protocol violation will be reported to the principal researcher. Appropriate treatments will be delivered to participants regarding their condition without delayed. Blinded allocation will be revealed if knowing of allocated intervention is important.

**Ethics**

This study will be conducted following the Declaration of Helsinki, the Belmont Report, and Godd Clinical Practice (GCP) guideline. The Institutional Review Board (IRB) of the Faculty of Medicine Vajira Hospital has approved this study (COA237/2564) before the first participant enrolment.

**Dissemination**

Results from this study will be disseminated via a peer-reviewed journal. No data that can identify individual participants will appear in the manuscript.

**References**

1. Yi JM, Bang JY, Choi B, Cho C, Lee YH, Lee EK, et al. Population-based volume kinetics of crystalloids and colloids in healthy volunteers. *Sci Rep* 2019;9(1):18638.
2. Lamke LO, Liljedahl SO. Plasma volume expansion after infusion of 5%, 20% and 25% albumin solutions in patients. *Resuscitation* 1976;5(2):85-92.
3. Park CH, Paik WH, Park ET, Shim CS, Lee TY, Kang C, et al. Aggressive intravenous hydration with lactated Ringer's solution for prevention of post-ERCP pancreatitis: a prospective randomized multicenter clinical trial. *Endoscopy* 2018;50(4):378-385.
