# Supplementary figures and images for: High-volume lactated Ringer’s solution with human albumin versus standard-volume infusion as a prophylactic treatment for post-endoscopic retrograde cholangiopancreatography pancreatitis: randomized clinical trial
Source: BJS Open. 2025 Jan 21;9(1):zrae149. doi: 10.1093/bjsopen/zrae149 (PMC11749547; doi:10.1093/bjsopen/zrae149)

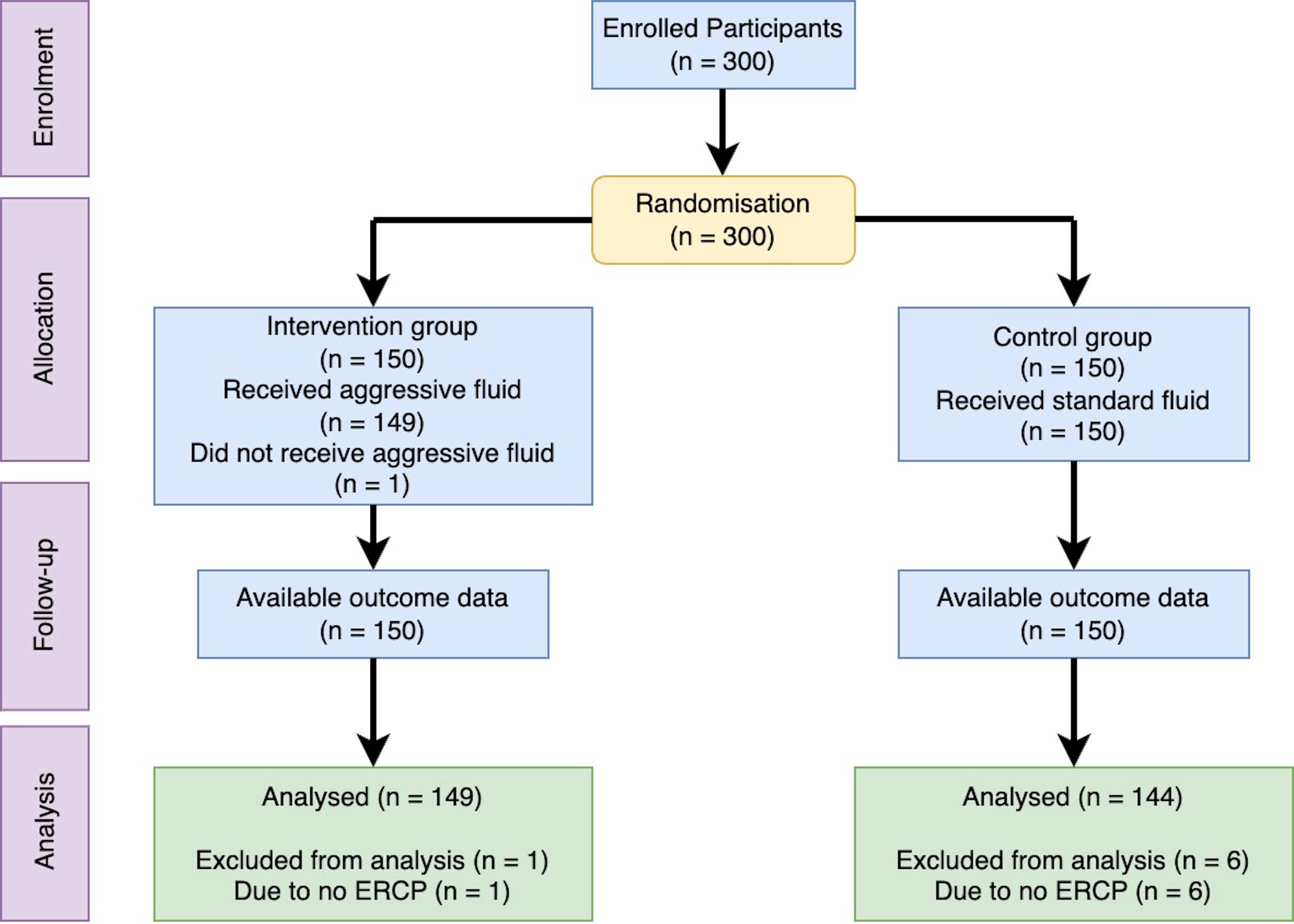

Supplement: zrae149_Supplementary_Data [file zrae149_supplementary_data.zip › modified_CONSORT-DIAGRAM.jpg]
